# Survival analysis and factors influencing survival time of adult HIV/AIDS patients receiving antiretroviral therapy from 2012 to 2022 in Chongqing, China: a retrospective cohort study

**DOI:** 10.3389/fpubh.2025.1614619

**Published:** 2025-09-19

**Authors:** Yao Huang, Ming He, Jinhua Zhang, Lihong Mu, Cheng Tang

**Affiliations:** ^1^Department of Non-communicable Chronic Disease Control and Prevention, Disease Control and Prevention Center of Jiulongpo Distract, Chongqing, China; ^2^Department of Epidemiology, School of Public Health, Research Center for Medicine and Social Development, Chongqing Medical University, Chongqing, China

**Keywords:** HIV/AIDS, survival analysis, prognosis, antiretroviral therapy, accelerated failure time model, cohort studies

## Abstract

**Background:**

Despite expanded antiretroviral therapy (ART) coverage in China, AIDS remains a major public health challenge. This study aimed to assess survival outcomes and identify factors influencing survival time among HIV/AIDS patients receiving ART.

**Methods:**

This study was a retrospective cohort study utilizing data on HIV patients receiving antiretroviral therapy in Jiulongpo District, Chongqing, from 2012 to 2022. Life tables estimated cumulative survival, Kaplan-Meyer compared survival between groups, and accelerated time to failure (AFT) modeling was used for analysis of factors influencing survival time.

**Results:**

The 1-, 3-, 5-, and 10-year cumulative survival rates were 94% (95% CI: 0.93 to 0.95), 90% (95% CI: 0.89 to 0.91), 86% (95% CI: 0.85 to 0.87), and 79% (95% CI: 0.76 to 0.81), respectively. Compared to patients aged 15–29, those aged 30–49 (TR = 0.22, 95% CI: 0.11 to 0.46, *p* < 0.01) and ≥50 (TR = 0.07, 95% CI: 0.03 to 0.15, *p* < 0.01) had shorter survival. Males (TR = 0.26, 95% CI: 0.17 to 0.38, *p* < 0.01), single (TR = 0.52, 95% CI: 0.33 to 0.84, *p* = 0.007), and divorced/widowed patients (TR = 0.67, 95% CI: 0.48 to 0.93, *p* = 0.018) had shorter survival compared to their counterparts. Infection via homosexual contact (TR = 0.21, 95% CI: 0.12 to 0.37, *p* < 0.01), injection drug use/other routes (TR = 0.09, 95% CI: 0.04 to 0.20, *p* < 0.01) was associated with shorter survival than heterosexual contact. Patients with tertiary (TR = 5.88, 95% CI: 3.05 to 11.33, *p* < 0.01), high school (TR = 2.86, 95% CI: 1.87 to 4.36, *p* < 0.01), and middle school education (TR = 1.93, 95% CI: 1.40 to 2.67, *p* = 0.002) had longer survival than those with primary/illiterate education. Baseline CD4 counts of ≥350 (TR = 3.26, 95% CI: 2.33 to 4.55, *p* < 0.01) and 200–349 cells/μL (TR = 2.73, 95% CI: 1.90 to 3.92, *p* < 0.01) were linked to longer survival compared to 0–199 cells/μL.

**Conclusion:**

Older age, males, lower educational attainment, homosexual contact, injection of drugs, and lower baseline CD4 levels are influential factors for shorter survival time in HIV/AIDS patients receiving ART. These findings may help inform clinical decision-making and targeted interventions to improve the long-term outcomes of people living with HIV.

## Introduction

AIDS is an immunodeficiency disease caused by HIV infection with predominantly defective immune function of T-lymphocytes, posing a major challenge to global public health. By the end of 2023, an estimated 39.9 million people were living with HIV worldwide, with 630,000 deaths from AIDS-related illnesses and 1.3 million new infections occurring that year. Anti-retroviral therapy (ART) is the main treatment strategy for curbing HIV infection, with 30.7 million people having access to ART, up from 7.7 million in 2010. However, this figure still falls short of the 2025 global target of 34 million, meaning that approximately 23% of people living with HIV are still not on treatment (1). This persistent treatment gap highlights the critical importance of conducting in-depth research on the survival status, treatment challenges, and influencing factors among people with HIV, especially within specific regional contexts, to provide evidence-based support for optimizing prevention and control strategies. The current status of the AIDS epidemic in China is also not optimistic, with 719,464 living HIV infections, 570,236 AIDS patients, and 457,609 deaths across China by the end of 2023 (2). Chongqing is located in southwestern China and is the largest municipality directly under the central government, as of October 2023 Chongqing has cumulatively reported 68,000 cases of HIV infection and 24,000 deaths, making it one of the more serious cities in China in terms of the AIDS epidemic (3).

Since ART was first proposed in 1996, the number of HIV/AIDS patients receiving ART has been growing globally, and ART has gradually transformed AIDS from a lethal disease into a chronic disease that can be effectively controlled by treatment and significantly extended the life expectancy of patients so that the life expectancy of HIV/AIDS patients is close to that of the general population (4–6). Starting from 2003, China began to implement the “Four Frees and One Care” policy for HIV-infected and AIDS patients, carried out free Highly Active Antiretroviral Therapy (HAART) nationwide. As a result, the cumulative number of ART patients has increased rapidly, the proportion of viral load suppression has steadily increased (7), and the mortality rate of AIDS patients has significantly decreased. Nevertheless, 457,609 AIDS-related deaths were reported by the end of 2023.

Extending the survival of patients receiving antiretroviral therapy (ART) remains a core focus of current HIV/AIDS prevention and control efforts. Despite the continuous expansion of ART coverage, the long-term risk of mortality and its associated factors continue to pose serious challenges in the field of public health. Conducting long-term, dynamic assessments of patient survival in specific regions is essential for developing targeted interventions and optimizing resource allocation. While some studies in China have examined the survival of ART patients, most have focused on other regions or covered shorter time spans, making them insufficient to capture the unique challenges faced by Chongqing, a major city in western China. Therefore, this study aims to address this critical gap in regional evidence by conducting, for the first time, a 10-year survival analysis (2012–2022) of an ART-treated cohort in Jiulongpo District, Chongqing, and exploring the factors associated with survival outcomes, with the goal of providing direct data support for improving patient care and informing local HIV/AIDS control strategies.

## Materials and methods

### Study design

This retrospective cohort study aimed to analyze the survival and its influencing factors among HIV/AIDS patients (aged ≥15 years at diagnosis) who were receiving or had received antiretroviral therapy (ART) in Jiulongpo District, Chongqing, China, from January 1, 2012, to December 31, 2022.

The start of follow-up was defined as the date of ART initiation. The end of follow-up was defined as the earliest of the following events: death from any cause, the date of the last recorded contact for patients lost to follow-up, or the administrative censoring date of December 31, 2022. Survival time was calculated as the interval, in years, from ART initiation to the end of follow-up.

All patients received ART in accordance with the Chinese Guidelines for the Diagnosis and Treatment of Human Immunodeficiency Virus Infection/Acquired Immunodeficiency Syndrome. The standard first-line regimen provided was a combination of Lamivudine (3TC), Efavirenz (EFV), and Tenofovir Disoproxil Fumarate (TDF). Final treatment decisions were individualized by the treating physician, taking into account the patient’s viral load, CD4 cell count, and overall drug tolerance. Patients were routinely followed up by clinicians and public health staff in local healthcare facilities in accordance with national guidelines: monthly during the first 2 months after ART initiation and every 3 months thereafter. Follow-up assessments included symptom evaluation, CD4 cell count testing, viral load monitoring, and evaluation of treatment adherence.

### Study subjects

The study data were obtained from the AIDS Prevention and Control Information System of the Chinese Center for Disease Control and Prevention. This study included individuals diagnosed with HIV infection or AIDS who received antiretroviral therapy (ART) between January 1, 2012, and December 31, 2022, in Jiulongpo District, Chongqing, China. The inclusion criteria were: (1) confirmed diagnosis of HIV infection or AIDS; (2) age ≥15 years at the time of diagnosis; (3) documented record of ART initiation; (4) living in Jiulongpo District, Chongqing. The exclusion criteria were: (1) no record of ART treatment; (2) missing baseline CD4 test results; (3) duplicate records or missing key information. Baseline information including gender, age, marital status, education level, transmission mode, and CD4 count was collected at the initiation of ART.

A total of 4,924 records of HIV/AIDS patients who initiated antiretroviral therapy (ART) between January 1, 2012, and December 31, 2022, in Jiulongpo District, Chongqing, were retrieved from the AIDS Prevention and Control Information System of the Chinese Center for Disease Control and Prevention. After applying the predefined inclusion and exclusion criteria, 898 cases were excluded. The final study population comprised 4,026 eligible patients.

### Quality control and ethical considerations

To ensure the quality of follow-up data, Disease Control and Prevention Center of Jiulongpo Distract, Chongqing regularly assessed data completeness, consistency, and logical accuracy. In addition, the Chongqing center for disease control and prevention (CDC) and the national CDC periodically conducted data quality audits and supervised the follow-up process to ensure accurate data collection and standardized procedures.

We verified and confirmed death information through the National Cause of Death Surveillance System, healthcare facility reports, and community visits. If a patient died during follow-up, the time of death was recorded; patients who were lost to follow-up before the end of the follow-up period and had no recorded death during the follow-up were treated as censored.

As staff of the Jiulongpo District Center for Disease Control and Prevention, we had authorized routine access to this database as part of our institutional public health responsibilities. Ethical approval for the analysis was granted by the Ethics Committee of the Jiulongpo District Center for Disease Control and Prevention, Chongqing.

### Statistical analysis

Data were organized and analyzed using R software version 4.3.2 (http://www.R-project.org, The R Foundation for Statistical Computing, Vienna, Austria). The chi-square test was used to describe the basic characteristics of the study population. Kaplan–Meier survival analysis was employed to estimate the probability of patient survival, and survival curves were plotted according to patient characteristics. The log-rank test was used to assess statistically significant differences in survival between groups. We also checked for the problem of multicollinearity between the variables by checking the variance inflation factor (VIF), which was examined and there was no multicollinearity between the variables. The proportional hazards (PH) assumption was tested using the Schoenfeld residuals method. As the data violated the PH assumption, as shown in Supplementary Table S1 and Supplementary Figure S1. An accelerated failure time (AFT) model was used instead of the Cox proportional hazards model to evaluate the effects of covariates on survival time (8, 9). The AFT model focuses on the acceleration or deceleration of survival time rather than the hazard ratio of death. A time ratio (TR) > 1 indicates that a higher level of the covariate is associated with prolonged survival time, whereas a TR < 1 indicates shortened survival time. All statistical tests were two-sided, and a *p*-value < 0.05 was considered statistically significant.

## Results

### Baseline characteristics

A total of 4,026 HIV/AIDS patients who received antiretroviral therapy (ART) from 2012 to 2022 were included in this study. The mean age of the participants was 44.64 ± 17.11 years, with 40.61% (*n* = 1,635) aged over 50 years. The majority of the patients were male (78.22%, *n* = 3,149). In terms of education level, 24.17% (*n* = 973) had no formal education or only elementary school, 28.84% (*n* = 1,161) had completed middle school, 22.33% (*n* = 899) had completed high school, and 24.66% (*n* = 993) had tertiary education. Regarding marital status, 46.99% (*n* = 1,892) were married. Most patients (66.89%, *n* = 2,693) were infected through heterosexual contact. The median baseline CD4 count was 296 (Q1:167, Q3:446) cells/μL. 31.50% (*n* = 1,268) had a baseline CD4 count <200 cells/μL, 27.89% (*n* = 1,123) had 200–350 cells/μL, and 40.61% (*n* = 1,635) had ≥350 cells/μL. Details are presented in Table 1.

**Table 1:** Demographic information of HIV/AIDS patients receiving ART in Jiulongpo District, Chongqing.

Characteristics	Total (*n* = 4,026)	Censored (*n* = 3,503)	Dead (*n* = 523)
Age (years)			
15–29	1,043 (25.91%)	1,015 (28.98%)	28 (5.35%)
30–49	1,348 (33.48%)	1,218 (34.77%)	130 (24.86%)
≥50	1,635 (40.61%)	1,270 (36.25%)	365 (69.79%)
Gender			
Male	3,149 (78.22%)	2,711 (77.39%)	438 (83.75%)
Female	877 (21.78%)	792 (22.61%)	85 (16.25%)
Education			
Primary and illiteracy	973 (24.17%)	735 (20.98%)	238 (45.51%)
Middle school	1,161 (28.84%)	992 (28.32%)	169 (32.31%)
High school	899 (22.33%)	818 (23.35%)	81 (15.49%)
Tertiary	993 (24.66%)	958 (27.35%)	35 (6.69%)
Marital status			
Married	1892 (46.99%)	1,594 (45.50%)	298 (56.98%)
Divorced	673 (16.72%)	548 (15.64%)	125 (23.90%)
Single	1,392 (34.58%)	1,309 (37.37%)	83 (15.87%)
Unknown	69 (1.71%)	52 (1.48%)	17 (3.25%)
Transmission mode			
Homosexual contact	1,194 (29.66%)	1,152 (32.89%)	42 (8.03%)
Heterosexual contact	2,693 (66.89%)	2,250 (64.23%)	443 (84.70%)
Injection of drugs and others	139 (3.45%)	101 (2.88%)	38 (7.27%)
CD4 count at baseline cells/μL			
0–199	1,268 (31.50%)	1,012 (28.89%)	256 (48.95%)
200–349	1,123 (27.89%)	1,010 (28.83%)	113 (21.61%)
≥350	1,635 (40.61%)	1,481 (42.28%)	154 (29.45%)

### Patient survival time

By the end of the observation period, a total of 523 all-cause deaths (13.00%) were recorded among the 4,026 HIV/AIDS patients over a total of 17,470.58 person-years of follow-up. This corresponds to an overall mortality rate of 2.99 per 100 person-years. The lost-to-follow-up rate was 0.82% (*n* = 33), and the mean follow-up time was 4.34 years.

**Figure 1:**
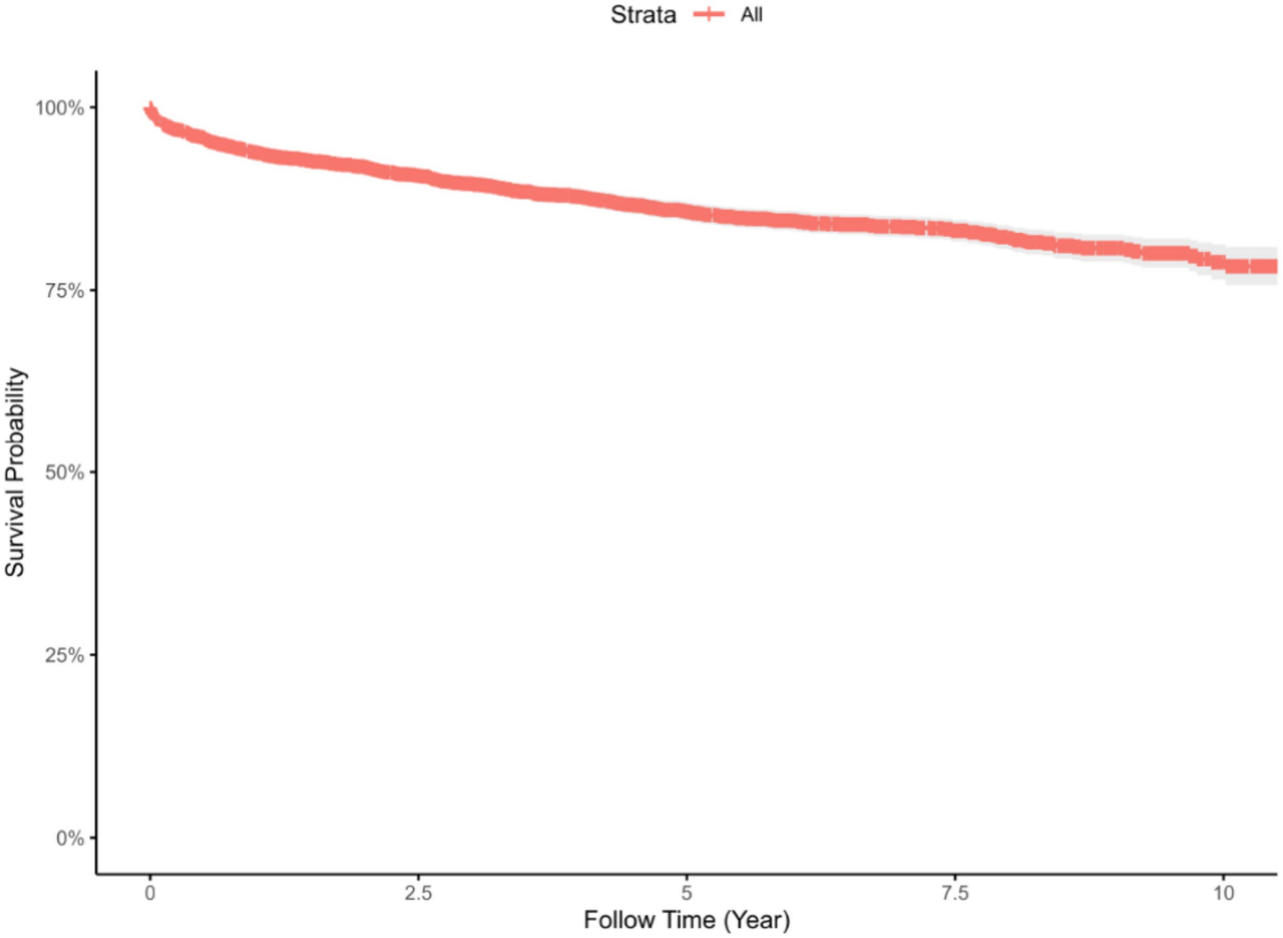
Survival curve for patients receiving ART in Jiulongpo District, Chongqing, from 2012 to 2022.

Survival analysis revealed that the highest number of deaths occurred during the first year after initiating ART (*n* = 242). The cumulative survival probabilities at 1-, 3-, 5-, and 10- year were 94% (95% CI: 0.93 to 0.95), 90% (95% CI: 0.89 to 0.91), 86% (95% CI: 0.85 to 0.87), and 79% (95% CI: 0.76 to 0.81), respectively. Median survival time was not reached during the follow-up period. Detailed results are presented in Table 2 and Figure 1.

**Table 2:** Cumulative survival rate of HIV/AIDS patients receiving ART from 2012 to 2022 in Jiulongpo District, Chongqing.

Time interval (year)	Number at risk (*n*)	Number of deaths (*n*)	Adjusted number at risk	Cumulative survival standard error	Interval mortality rate	Cumulative survival rate (95%CI)
1	4,026	242	3816.50	0.0039	0.063	0.94 (0.93 ~ 0.95)
2	3,365	65	3,150	0.0045	0.021	0.92 (0.90 ~ 0.93)
3	2,870	67	2,685	0.0052	0.025	0.90 (0.89 ~ 0.91)
4	2,433	44	2,242	0.0057	0.020	0.88 (0.87 ~ 0.89)
5	2007	43	1827.50	0.0064	0.024	0.86 (0.85 ~ 0.87)
6	1,605	22	1,440	0.0068	0.015	0.85 (0.83 ~ 0.86)
7	1,253	11	1082.50	0.0072	0.010	0.84 (0.82 ~ 0.85)
8	901	13	764.50	0.0082	0.017	0.82 (0.81 ~ 0.84)
9	615	9	503	0.0093	0.018	0.81 (0.79 ~ 0.83)
10	382	6	269.50	0.0122	0.022	0.79 (0.76 ~ 0.81)

**Figure 2:**
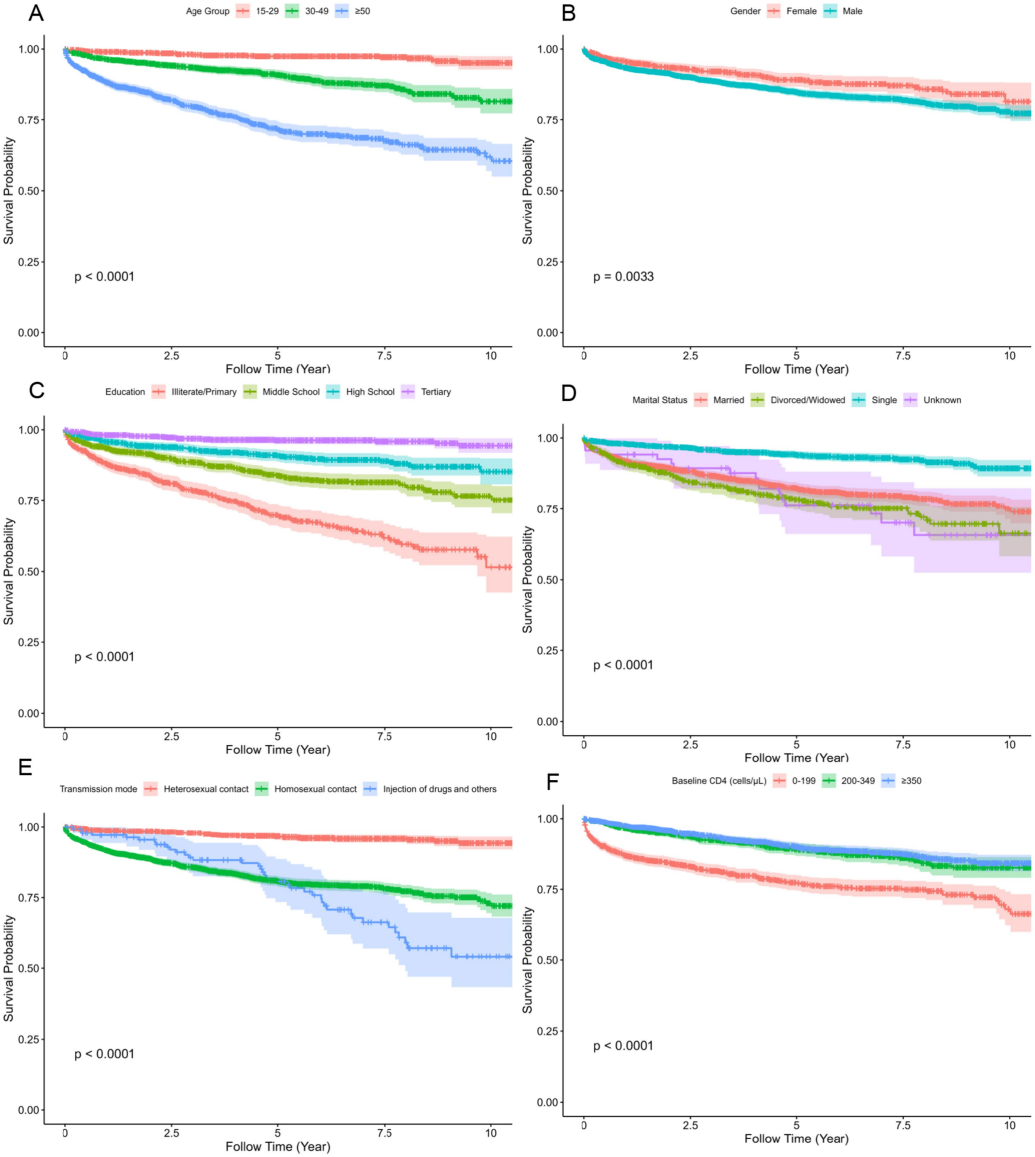
Kaplan–Meier survival curves showing differences in cumulative survival among subgroups of adult HIV/AIDS patients on ART in Jiulongpo District, Chongqing, China, 2012–2022. The panels show survival probabilities for patients grouped by **(A)** age group, **(B)** gender, **(C)** education, **(D)** marital status; **(E)** transmission mode, and **(F)** baseline CD4 count. The *p*-values for the comparison of survival distributions among groups were calculated using the log-rank test.

Kaplan–Meier survival curves showed that patients aged ≥50 years had the lowest survival probability (*p* < 0.001). Male patients had significantly lower survival probability compared to females (*p* = 0.003). Education level was associated with survival outcomes, with the lowest and fastest-declining survival curves observed among those with only primary and illiteracy (*p* < 0.001). Patients infected through injection drug use and other transmission routes exhibited the poorest survival outcomes (*p* < 0.001). Higher baseline CD4 counts were associated with better survival, with patients having ≥350 cells/μL demonstrating the highest probability of survival (*p* < 0.001), as shown in Figure 2.

### Analysis of factors affecting patient survival time

The results of the univariable AFT model showed that age, gender, education level, and baseline CD4 level were all influencing factors on the survival time of the study subjects, and the difference was statistically significant (*p* < 0.05).

The results of the multivariate AFT model showed that older age was significantly associated with shorter survival time from the initiation of ART follow-up to death. Compared to patients aged 15–29 years, those aged 30–49 years and ≥50 years had survival times that were 0.22 times (95% CI: 0.11 to 0.46, *p* < 0.01) and 0.07 times (95% CI: 0.03 to 0.15, *p* < 0.01) as long, respectively. Male patients had significantly shorter survival times than female patients (TR = 0.26, 95% CI: 0.17 to 0.38, *p* < 0.01). Higher education levels were associated with longer survival. Patients with tertiary education, high school education, and middle school education had survival times that were 5.88 times (95% CI: 3.05 to 11.33, *p* < 0.01), 2.86 times (95% CI: 1.87 to 4.36, *p* < 0.01), and 1.93 times (95% CI: 1.40 to 2.67, *p* = 0.002) those of patients with primary school or no formal education, respectively. Marital status also significantly influenced survival. Compared to married individuals, divorced/widowed (TR = 0.67, 95% CI: 0.48 to 0.93, *p* = 0.018), single (TR = 0.52, 95% CI: 0.33 to 0.84, *p* = 0.007), and those with unknown marital status (TR = 0.40, 95% CI: 0.18 to 0.89, *p* = 0.025) had shorter survival times. Regarding the transmission mode, patients infected through homosexual contact (TR = 0.21, 95% CI: 0.12 to 0.37, *p* < 0.01) or injection drug use and other routes (TR = 0.09, 95% CI: 0.04 to 0.20, *p* < 0.01) had significantly shorter survival times compared to those infected through heterosexual contact. Baseline CD4 count was also strongly associated with survival. Compared to patients with baseline CD4 counts of 0–199 cells/μL, those with counts of 200–349 cells/μL and ≥350 cells/μL had survival times that were 4.26 times (95% CI: 2.85 to 6.37, *p* < 0.01) and 4.99 times (95% CI: 3.45 to 7.22, *p* < 0.01) longer, respectively as shown in Table 3.

**Table 3:** Analysis of factors affecting survival time of HIV/AIDS patients receiving ART in Jiulongpo District, Chongqing.

Variable	Patient (*n*)	Survival time (years)	Univariate model		Multivariate model	
			TR (95%CI)	*p*-value	ATR (95%CI)	*P*-value
Age						
15–29	1,043	5.62 (3.23–7.95)	1.00 (Ref)		1.00 (Ref)	
30–49	1,348	4.44 (2.11–6.97)	0.10 (0.05 ~ 0.20)	<0.01	0.22 (0.11 ~ 0.46)	<0.01
≥50	1,635	2.52 (0.99–5.04)	0.02 (0.01 ~ 0.03)	<0.01	0.07 (0.03 ~ 0.15)	<0.01
Gender						
Female	877	3.89 (1.46–6.49)	1.00 (Ref)		1.00 (Ref)	
Male	3,149	3.98 (1.69–6.69)	0.54 (0.36 ~ 0.82)	0.003	0.26 (0.17 ~ 0.38)	<0.01
Education						
Primary and illiteracy	973	2.43 (0.99–4.85)	1.00 (Ref)		1.00 (Ref)	
Middle school	1,161	3.92 (1.43–6.57)	3.30 (2.35 ~ 4.63)	<0.01	1.93 (1.40 ~ 2.67)	0.002
High school	899	4.68 (2.07–7.17)	8.57 (5.49 ~ 13.38)	<0.01	2.86 (1.87 ~ 4.36)	<0.01
Tertiary	993	4.85 (2.61–7.42)	44.09 (22.98 ~ 84.56)	<0.01	5.88 (3.05 ~ 11.33)	<0.01
Marital status						
Married	1892	3.49 (1.39–6.44)	1.00 (Ref)		1.00 (Ref)	
Divorced/Widowed	673	3.36 (1.17–5.66)	0.67 (0.46 ~ 0.96)	0.028	0.67 (0.48 ~ 0.93)	0.018
Single	1,392	4.73 (2.48–7.37)	6.87 (4.41 ~ 10.70)	<0.01	0.52 (0.33 ~ 0.84)	0.007
Unknown	69	5.42 (3.44–7.62)	0.64 (0.28 ~ 1.51)	0.307	0.40 (0.18 ~ 0.89)	0.025
Transmission mode						
Heterosexual contact	1,194	5.25 (2.93–7.69)	1.00 (Ref)		1.00 (Ref)	
Homosexual contact	2,693	3.36 (1.26–6.10)	0.05(0.03 ~ 0.09)	<0.01	0.21 (0.12 ~ 0.37)	<0.01
Injection of drugs and others	139	5.21 (2.41–7.80)	0.03(0.01 ~ 0.06)	<0.01	0.09 (0.04 ~ 0.20)	<0.01
CD4 count at baseline cells/μL						
0–199	1,268	3.06 (1.06–5.75)	1.00 (Ref)		1.00 (Ref)	
200–349	1,123	3.92 (1.89–6.68)	4.37 (2.94 ~ 6.50)	<0.01	2.73 (1.90 ~ 3.92)	<0.01
≥350	1,635	4.72 (2.11–7.14)	5.30 (3.68 ~ 7.63)	<0.01	3.26 (2.33 ~ 4.55)	<0.01

TR, time ratio; ATR, adjust time ratio.

Ref, reference category.

Survival time: represents the median (Interquartile Range, IQR) of the observed follow-up duration for each subgroup.

## Discussion

In the baseline description, the mean age of the study population was 44.64 ± 17.11 years, with 40.61% of patients over the age of 50. A study in the United States projected that by 2023, 23% of individuals receiving ART would be aged 65 years or older (10). With the widespread adoption and increasing effectiveness of ART, the aging trend among people living with HIV/AIDS (PLWHA) has become increasingly prominent. This shift necessitates greater attention from healthcare systems and researchers to address the unique characteristics and care needs of older HIV-infected individuals, including tailored interventions to improve their health outcomes and quality of life. The high proportion of male patients in our study (78.22%) may be partly attributed to men who have sex with men (MSM) as a key route of HIV transmission. Even among heterosexual populations, men are often the dominant or more active party in sexual activity and may be more frequently exposed to high-risk partners, thereby increasing their infection risk (11). Heterosexual transmission is the main mode of HIV transmission in the region, and a report from the China Medical University states that heterosexual transmission is the main mode of transmission in southwestern China, accounting for 50–70% of new cases (12). In Southwest China, casual sex and multiple partner relationships are more common, which increases the risk of HIV transmission, and studies in Chongqing have also shown that heterosexual transmission has become the main mode of HIV transmission in the region (13). In addition, homosexual behavior is usually hidden and drug use is illegal in China, making it difficult to obtain true reports in surveys, so the actual proportion of transmission through MSM and injecting drug use in the available data may be lower than the true level.

HIV/ADIS patients receiving ART performed well in terms of long-term survival, with survival analyses showing 1st, 3rd, 5th, and 10th year cumulative survival rates of 94% (95% CI: 0.93 to 0.95), 90% (95% CI: 0.89 to 0.91), 86% (95% CI: 0.85 to 0.87), and 79% (95% CI: 0.76 to 0.81), which differed from the rest of China and were higher than those in less developed western regions such as Gansu, Liangshan Prefecture in Sichuan, and Guizhou (14–16), but slightly lower than those in economically developed cities such as Beijing and Nanjing (17, 18), which may be related to the differences in medical resource allocation, patient education level and quality of life in different regions. We found that the highest number of new deaths (*n* = 242) occurred among HIV/AIDS patients who received ART in the first year, suggesting that the initial treatment period is a critical stage for patient survival. Although ART significantly reduces overall mortality, it remains high in resource-limited settings, especially in the first few months after treatment initiation (19–21). The main causes of death include tuberculosis, acute bacterial infections, malignancies and immune reconstitution disorders (22), in addition to poor early treatment adherence, which increases the risk of early death (23), Many early deaths occur in patients with advanced immunodeficiency who have not yet started ART, further emphasizing the importance of increased follow-up and monitoring of newly treated patients, improving treatment adherence, and supporting immune system reconstitution.

The results of the multivariate AFT model showed that patients aged 30–49 years and those over 50 years had shorter survival times compared to those aged 15–29 years. In addition to age itself being a key factor influencing survival, older patients often suffer from multiple chronic diseases, immune system decline, and various comorbidities, which accelerate HIV progression (24), thereby reducing the effectiveness of ART. Female patients exhibited longer survival times than males, consistent with findings from studies in India and Tanzania (25, 26). This may be due to biological differences in pharmacokinetics and immune responses between the sexes (27), with regard to social factors, socially, women may be more likely to seek medical assistance, follow medical advice, and adhere to treatment regimens, which could contribute to improved survival outcomes. We also found that education was a key factor in survival time, and Nanjing reported similar results (18). Highly educated patients may be more concerned about their health, as well as having easier access to information about healthcare services, and in addition, highly educated patients may be in a better financial situation and have access to better healthcare resources. In terms of marital status, single patients appeared to have longer survival in univariate analysis (TR = 6.87, 95% CI: 4.41 to 10.70); however, this advantage disappeared in the multivariate model (TR = 0.52, 95% CI: 0.33 to 0.84). Upon further examination of the sample composition, we found that 86.48% of single patients were aged 15–29 years, suggesting that the survival advantage was mainly driven by younger age. After adjusting for age, single status itself was associated with shorter survival time. This indicates that being single may be an independent risk factor, as single patients often face greater socioeconomic challenges, lack stable income sources, and may struggle to afford medical care or maintain a healthy lifestyle (28). Social support plays a crucial role in ART adherence; single individuals may lack the support of a partner, family, or close social networks, which can negatively impact their mental health, treatment adherence, and ultimately, survival (29). Patients infected through homosexual contact had shorter survival times compared to those infected through heterosexual transmission, consistent with findings from studies conducted in the Caribbean and Latin American regions (30). This disparity is likely multifactorial. On one hand, the MSM community faces multiple challenges in terms of health awareness and access to healthcare services. Social discrimination and stigmatization can hinder early detection and treatment, as some may conceal their sexual orientation for fear of compromising their privacy (31). On the other hand, beyond these socio-behavioral barriers, virological factors may also play a significant role. Evidence suggests that the distribution of HIV-1 subtypes can differ among risk groups, with certain circulating recombinant forms (CRFs), such as CRF01_AE, being more prevalent in MSM populations, particularly in Asia (32). These specific genotypes have been associated with higher virulence and a more rapid decline in CD4 + T-cell counts, potentially leading to faster disease progression (33). Therefore, it is plausible that the survival disadvantage observed in our MSM group results from a complex interplay between social challenges and the underlying molecular epidemiology of the virus. Similarly, patients infected through injecting drug use had significantly shorter survival times than those infected via heterosexual transmission, echoing findings from previous studies (15, 29, 34, 35). Injecting drug users often engage in high-risk behaviors such as needle sharing, which not only increases the risk of HIV infection but also exposes them to co-infections like hepatitis B and C. These co-infections can further compromise immune function and reduce ART effectiveness (36). Moreover, chronic drug abuse can cause malnutrition, liver impairment and immunosuppression, resulting in a systemic double whammy on immunization (37). Treatment adherence is typically lower among drug-using populations. Drug dependence itself can interfere with medication routines, while frequent relocation, incarceration, and social instability make it difficult for this group to receive continuous follow-up and standardized ART. These disruptions increase the risk of treatment interruption and viral resistance. Socially, drug users are often subjected to stigma and family estrangement, and many lack basic social support systems. Additionally, hidden identities and financial hardship may prevent them from accessing timely medical care (38), further affecting their health outcomes. These combined unfavorable factors contribute to the fact that survival time after ART remains low among people infected with injecting drug use. Patients with higher baseline CD4 levels had longer survival times, and Kaplan–Meier curves also demonstrated a higher probability of survival for patients with higher baseline CD4 counts. CD4 directly reflects the body’s immune competence, and baseline CD4 counts and viral loads are important markers of survival, with lower CD4 cell counts tending to correlate with a poorer prognosis (35), and the results of several studies have shown that the lower the baseline CD4 counts, the worse the prognosis of patients after initiating ART (39–41), and overall CD4 cell counts increase significantly in HIV/AIDS patients treated with ART, reflecting rebuilding of the immune system and restoration of function. However, specific CD4 cell count recovery may vary depending on individual differences, treatment regimen, and baseline CD4 levels.

This study possesses several significant strengths that underscore the validity and importance of its findings. It is distinguished by a large, population-based design that includes all 4,026 eligible patients from an entire district over a decade, effectively minimizing the selection bias common in single-center or hospital-based studies. This design ensures the findings are highly representative of the real-world patient population, which enhances external validity and generalizability. The substantial sample size also provides high statistical power. The decade-long follow-up period offers a rare and valuable opportunity to analyze long-term survival outcomes, providing insights that are more clinically meaningful for a chronic, manageable condition like HIV/AIDS and capturing the full patient trajectory on ART. The study also demonstrates strong methodological rigor, with mortality status carefully verified through multiple official and community channels to improve the accuracy of the primary outcome. In addition, the use of an Accelerated Failure Time (AFT) model, chosen because the Proportional Hazards assumption was violated, ensures a more appropriate and robust statistical analysis and increases confidence in the reported associations. Finally, the study addresses a critical evidence gap by providing the first long-term survival analysis among ART recipients in Jiulongpo District, Chongqing. The localized evidence generated by this research can directly inform targeted, region-specific HIV/AIDS prevention and control strategies, offering practical value for healthcare providers and policymakers working to improve patient survival in this high-burden setting.

This study also has limitations. It did not differentiate specific causes of death and lacked both HIV-negative controls and untreated HIV/AIDS patient controls. The analysis was constrained by the variables available in the national database, preventing inclusion of important predictors such as viral load, adherence behavior, comorbid conditions (e.g., tuberculosis, hepatitis C), and lifestyle factors (e.g., smoking, alcohol use), all of which are known to influence survival outcomes. These missing data may have introduced unmeasured confounding and limit the interpretation and generalizability of the results. Future research incorporating these variables will be essential for a more comprehensive understanding of survival determinants among people living with HIV.

## Conclusion

In conclusion, we observed a higher risk of death within the first year of treatment in patients receiving ART, suggesting the need for better monitoring and risk management in the early stages of treatment. Older adults patients, males, those with lower education levels, individuals who transmit infections through homosexual contact, injecting drug use and those with baseline CD4 counts below 200 cells/μL are high-risk groups for shorter survival times. Health organizations should pay more attention to these groups, develop targeted interventions, and strengthen risk assessment and management to improve treatment outcomes and patient prognosis.

## Data Availability

The raw data supporting the conclusions of this article will be made available by the authors, without undue reservation.
