# Supplementary material for: Survival analysis and factors influencing survival time of adult HIV/AIDS patients receiving antiretroviral therapy from 2012 to 2022 in Chongqing, China: a retrospective cohort study
Source: Front Public Health. 2025 Sep 19;13:1614619. doi: 10.3389/fpubh.2025.1614619 (PMC12491286; doi:10.3389/fpubh.2025.1614619)
Supplement: Supplementary file 1 [file Data_Sheet_1.pdf]

Supplementary Material

Table S1. Results of the Proportional Hazards Assumption Test for Covariates in the Multivariable Cox Model.

**Variables**	**df**	χ^2	***P value***
Age group	2	2.79	0.25
Gender	1	0.016	0.90
Marital status	3	5.802	0.12
Education	3	4.21	0.18
Transmission mode	2	25.45	<0.01
Baseline CD4 count	2	47.08	<0.01
Global	13	73.197	<0.01

The plots show the scaled Schoenfeld residuals against time for the two covariates that significantly violated the PH assumption. Transmission mode ($P < 0.01$); Baseline CD4 count ($P < 0.01$). The solid line represents the smoothed estimate of the residual trend, with the dashed lines indicating the 95% confidence interval. A non-zero slope in the trend line suggests a deviation from the proportional hazards assumption.

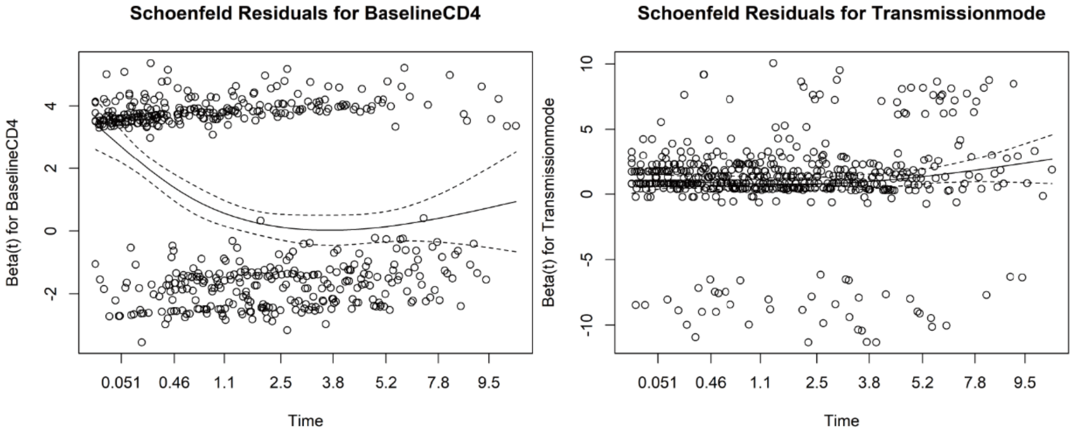

Figure S1. Plots of Scaled Schoenfeld Residuals for Covariates Violating the Proportional Hazards Assumption.
